# Redefining the Chronic-Wound Microbiome: Fungal Communities Are Prevalent, Dynamic, and Associated with Delayed Healing

**DOI:** 10.1128/mBio.01058-16

**Published:** 2016-09-06

**Authors:** Lindsay Kalan, Michael Loesche, Brendan P. Hodkinson, Kristopher Heilmann, Gordon Ruthel, Sue E. Gardner, Elizabeth A. Grice

**Affiliations:** a Department of Dermatology, Perelman School of Medicine, University of Pennsylvania, Philadelphia, Pennsylvania, USA; b Department of Microbiology, Carver College of Medicine, University of Iowa, Iowa City, Iowa, USA; c College of Nursing, University of Iowa, Iowa City, Iowa, USA; d PennVet Imaging Core, School of Veterinary Medicine, University of Pennsylvania, Philadelphia, Pennsylvania, USA; e Department of Microbiology, Perelman School of Medicine, University of Pennsylvania, Philadelphia, Pennsylvania, USA

## Abstract

Chronic nonhealing wounds have been heralded as a silent epidemic, causing significant morbidity and mortality especially in elderly, diabetic, and obese populations. Polymicrobial biofilms in the wound bed are hypothesized to disrupt the highly coordinated and sequential events of cutaneous healing. Both culture-dependent and -independent studies of the chronic-wound microbiome have almost exclusively focused on bacteria, omitting what we hypothesize are important fungal contributions to impaired healing and the development of complications. Here we show for the first time that fungal communities (the mycobiome) in chronic wounds are predictive of healing time, associated with poor outcomes, and form mixed fungal-bacterial biofilms. We longitudinally profiled 100, nonhealing diabetic-foot ulcers with high-throughput sequencing of the pan-fungal internal transcribed spacer 1 (ITS1) locus, estimating that up to 80% of wounds contain fungi, whereas cultures performed in parallel captured only 5% of colonized wounds. The “mycobiome” was highly heterogeneous over time and between subjects. Fungal diversity increased with antibiotic administration and onset of a clinical complication. The proportions of the phylum *Ascomycota* were significantly greater (*P* = 0.015) at the beginning of the study in wounds that took >8 weeks to heal. Wound necrosis was distinctly associated with pathogenic fungal species, while taxa identified as allergenic filamentous fungi were associated with low levels of systemic inflammation. Directed culturing of wounds stably colonized by pathogens revealed that interkingdom biofilms formed between yeasts and coisolated bacteria. Combined, our analyses provide enhanced resolution of the mycobiome during impaired wound healing, its role in chronic disease, and impact on clinical outcomes.

## IMPORTANCE

Wounds are an underappreciated but serious complication for a diverse spectrum of diseases. High-risk groups, such as persons with diabetes, have a 25% lifetime risk of developing a wound that can become chronic. The majority of microbiome research related to chronic wounds is focused on bacteria, but the association of fungi with clinical outcomes remains to be elucidated. Here we describe the dynamic fungal communities in 100 diabetic patients with foot ulcers. We found that communities are unstable over time, but at the first clinical presentation, the relative proportions of different phyla predict healing times. Pathogenic fungi not identified by culture reside in necrotic wounds and are associated with a poor prognosis. In wounds stably colonized by fungi, we identified yeasts capable of forming biofilms in concert with bacteria. Our findings illuminate the associations of the fungal mycobiome with wound prognosis and healing.

## INTRODUCTION

In recent years, the implication of microorganisms in complex human processes has begun to come into focus. Negative consequences of these interactions can result in large health care burdens such as nonhealing or chronic wounds (1–5). One example is diabetic-foot ulcers (DFUs), which contribute to 80% of nontraumatic lower-extremity amputations and are associated with 5-year mortality rates of 43 to 55%, higher than Hodgkin’s disease, breast cancer, or prostate cancer (6–8). Chronic wounds are largely believed to be critically colonized by polymicrobial communities that contribute to persistent inflammation and stalled healing processes, significantly reducing the quality of life for those afflicted (9–11). Skin normally harbors diverse communities of microbes (12–17) that can contribute to health, but like other ecosystems, the niche can direct composition and ultimately function (18–20). In tissue injury, microbes enter the wound where the physical environment differs from the skin surface in temperature, pH, nutrient availability, and host immune effectors. Here, microbial metabolism can shift, providing opportunities for commensal microbes to become virulent and community composition to fluctuate in response to host clinical factors (21–23). It is hypothesized that once colonization occurs, these communities form a biofilm within the wound, disrupting the coordinated tissue regeneration process. This is also true in other chronic infections, such as cystic fibrosis (CF) where the environment of the lungs in CF patients allows for colonization (24), unlike the lungs in healthy individuals, which can clear microbes.

Prior research has primarily focused on the role of bacterial species in wound healing (10, 23, 25–30); however, skin is also host to resident fungi, and our environment is rich with fungal diversity (31–34). Many human commensal fungi or yeasts are also opportunistic pathogens, and many species are known to be prolific biofilm formers (34–38). There are few studies describing the “mycobiome” portion of the human microbiome and its relation to health (32, 34, 39–41), while the incidence of fungal colonization in chronic wounds is even less known. A previous cross-sectional study of chronic wounds of mixed etiology and without standardized treatment utilized molecular-biology-based methods to observe that up to 23% of chronic wounds contain fungi (41). Chellan et al. studied 518 diabetic lower leg wounds and detected fungi in 27% of samples with culture-based methodology (42). In these studies, several aspects of the cross-sectional study design limit the ability to draw conclusions. These designs preclude longitudinal observation of fungal colonization and the relationship to clinical outcomes (i.e., rate of healing, infection-related complications), while controlling for clinical variables, such as tissue perfusion or blood glucose control.

Here, we add a new perspective to the current models of impaired wound healing with a longitudinal study of 100 DFUs under standardized treatment. High-throughput sequencing of the nuclear ribosomal internal transcribed spacer 1 (ITS1) allowed us to define the dynamic diversity of the mycobiome, its stability in response to host factors, and the association of pathogenic fungi with poor clinical outcomes.

## RESULTS

### Study overview.

To minimize variability associated with wound etiology, we limited our study to a single wound etiology consisting solely of DFUs. Subjects were enrolled in the study, and wound specimens were obtained every 2 weeks until the wounds healed, another infection occurred, the wounds resulted in amputation, or the wounds were not closed after 26 weeks (visit 0 to 12). Table 1 summarizes the cohort where a total of 384 specimens were collected from 100 DFUs in a sample of 100 subjects. Additional clinical factors measured include white blood cell (WBC) count, ankle-brachial index (ABI), toe-brachial index (TBPI), hemoglobin A1c, glucose (HgbA1c), C-reactive protein, and transcutaneous oxygen levels of the wound edge. Monofilament testing confirmed neuropathy in all subjects. Complications were experienced by 31 (31%) subjects and were defined as follows: (i) wound deterioration, (ii) development of osteomyelitis, and/or (iii) amputation.

**TABLE 1:** Subject demographics and wound characteristics

Characteristic	No. of subjects with characteristic or parameter value
Subjects	100
Specimens	384
Sex (male/female)	78/22
Type 2 diabetes	87
Ulcer duration, wk [mean (SD)]	33.1 (41.6)
Ulcer location	
Forefoot	73
Midfoot	20
Heel	7
End of study outcome	
Healed	75
Unhealed	5
Amputation	7
Other infection	3
Dropped study	10
Subjects with detected ITS1	79
Specimens with detected ITS1	275

The fungal component of DFU microbiomes was studied by sequencing the hypervariable internal transcribed spacer 1 (ITS1) region of the eukaryotic rRNA cistron using the Illumina MiSeq platform (two 300-bp paired-end [PE] chemistry). The ITS region has been formally recognized as the universal barcode for fungal identification (43) so we elected to use this region and the curated fungal bar code reference database UNITE (44) for operational taxonomic unit (OTU) assignment. We employed the PIPITS pipeline (45) because it extracts the ITS subregion from raw reads and assigns taxonomy with a trained RDP Classifier (46). Of the 10,673,363 sequences, 10,593,779 sequences were identified as containing an ITS1 subregion. DNA from *Saccharomyces cerevisiae* was detected in the medium in which the wound samples were collected, and therefore, all OTUs identified at the genus or species level as *Saccharomyces* were filtered from the data set, resulting in removal of three phylotypes. After quality filtering and contaminant removal, 2,842,822 reads remained, resulting in 482 OTUs, and taxonomic identification yielded 284 phylotypes.

### Characterization of the DFU fungal mycobiome.

**FIG 1:**
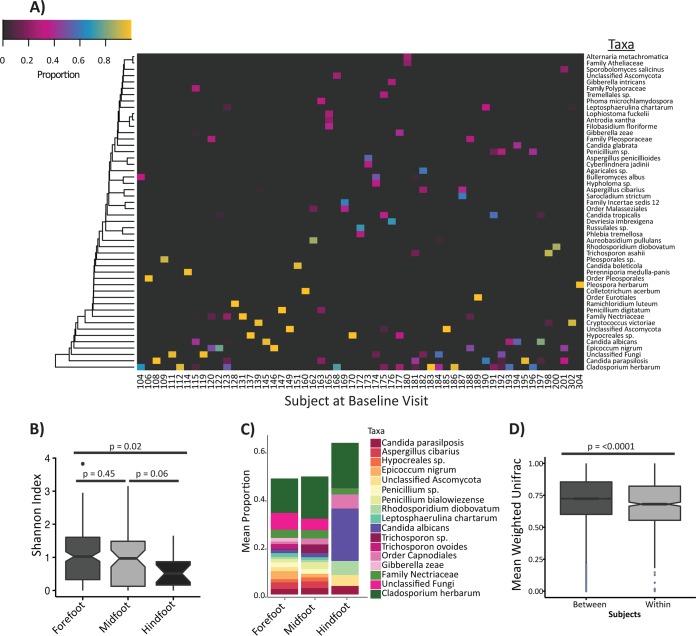
The DFU mycobiome is diverse and highly heterogeneous. (A) Heatmap of fungal community structure for all subjects at the baseline study visit. Hierarchical clustering was performed for taxa found in >20% abundance in at least one specimen. (B) Boxplot showing the Shannon diversity index (*y* axis) by wound location (*x* axis). *P* values were calculated by pairwise Wilcoxon rank sum test and adjusted for multiple comparison by the method of Holm. (C) Relative abundance plot showing the mean proportions of taxa found in >1% abundance in all of the samples (*y* axis) by wound location (*x* axis). Taxa found in significantly different abundance in the forefoot and hind foot wounds are listed in Table S2 in the supplemental material. (D) Boxplot showing the weighted UniFrac distances (*y* axis) between subjects (baseline study visit only) and within subjects longitudinally (*x* axis). The *P* value was calculated by Wilcoxon rank sum test. Notches in boxplots display the 95% confidence interval around the median.

Seventeen phylotypes were identified at a relative abundance of >1% across the entire data set, all belonging to the phylum *Ascomycota* or *Basidiomycota*. The two most abundant species, both belonging to the phylum *Ascomycota*, were *Cladosporidium herbarum* (teleomorph *Davidiella tassiania*), present in 41% of the samples and 56% of subjects, and *Candida albicans* (22% of samples and 47% of subjects). Notably, 10 of the 17 most abundant taxa are *Ascomycota* filamentous fungi found ubiquitously in the environment, while the most abundant *Basidiomycota* identified were the opportunistic yeast pathogens *Trichosporon* and *Rhodosporidium* spp. (Table 2 and Fig. 1A).

**TABLE 2:** Distribution of fungal taxa in >1% abundance

Fungal taxon	% specimens	No. of subjects
*Ascomycota*		
*Cladosporium herbarum*	41	56
*Candida albicans*	22	47
Unclassified *Ascomycota*	16	36
Family *Nectriaceae*	16	41
*Candida parapsilosis*	15	37
*Aspergillus cibarius*	12	30
*Epicoccum nigrum*	9	27
*Penicillium* sp.	9	26
*Leptosphaerulina chartarum*	7	23
*Penicillium bialowiezense*	6	19
*Gibberella zeae*	6	18
*Hypocreales* sp.	4	14
Order *Capnodiales*	4	15
*Basidiomycota*		
*Trichosporon asahii*	10	24
*Trichosporon* sp.	4	12
*Rhodosporidium diobovatum*	5	16
Unclassified fungi	32	55

*Malassezia* species are reported as a major component of the healthy skin mycobiome (32) and were detected at an abundance of >0.05% in 26 subjects in a total of 36 specimens, but only seven specimens had >10% relative abundance of *Malassezia* species. This observation is not unexpected, as *Malassezia* spp. are lipid dependent, and the foot is known to have overall lower abundances of *Malassezia* spp. relative to other body site niches (32, 47), in part due to absence of sebum in this microenvironment and in the wound itself.

A major current limitation of molecular DNA sequencing-based analysis of the mycobiota is the representation of fungal species in available reference databases. It is estimated that sequence data are available for a mere 1.5% of the estimated 1.5 million fungal species (46). Indeed, 32% of the DFU samples contained OTUs that could not be assigned beyond the kingdom level, and 16% of samples contained OTUs unclassified beyond phylum *Ascomycota* (Table 2).

Limitations of reference databases necessitate the use of reference-independent analyses to characterize diversity and variation of fungal communities. Alpha diversity metrics use reference-independent OTU data to summarize diversity, such as the number of OTUs in a sample and their evenness. We analyzed DFU mycobiome alpha diversity with respect to wound health and inflammation factors measured at the patient and ulcer level (see Fig. S1 and Table S1 in the supplemental material). At the first or baseline visit (visit 0), diversity measured as the number of observed species-level OTUs (median, 6; range, 1 to 21) and Faith’s phylogenetic distance (PD) (median, 4.64; range, 1.31 to 14.92) was negatively associated with tissue oxygenation (rho = −0.258 and *P* = 0.046 and rho = −0.295 and *P* = 0.022, respectively) (Fig. S1), suggesting that high fungal diversity is associated with poor perfusion.

As measured by the Shannon diversity index, which takes into account both richness and evenness of OTUs, ulcers on the forefoot were more diverse (median, 1.03; range, 0 to 3.83) than ulcers on the hind foot (median, 0.51; range, 0 to 1.65) (*P* = 0.02 by Wilcoxon rank sum test) with midfoot ulcers falling in between (median, 0.95; range, 0 to 3.15) to create a gradient of diversity (Fig. 1B). The number of observed species-level OTUs did not differ significantly between the forefoot and hind foot, indicating that the evenness of differentially abundant OTUs contributes to topographical variability of the mycobiome. Decreased Shannon diversity appears to be driven primarily by a significant increase in the relative abundance of *C. albicans* in ulcers formed on the hind foot (Fig. 1C; see Table S2 in the supplemental material).

At each study visit, wound specimens were also collected for quantitative cultures. Three subjects were culture positive for yeast isolates categorized as “skin flora” or “other,” and two subjects were positive for *Candida* sp. at one study visit. In all five cases, the study visit positive for yeast culture was concurrent with or preceded the study visit where a complication was observed (see Fig. S2 in the supplemental material). With a culture-independent sequence-based approach, after quality and contaminant filtering, 275 of the 384 specimens analyzed, corresponding to 79 subjects, were positive for at least one fungal phylotype (Table 1). The culture-positive specimens were confirmed by ITS1 sequencing to contain high relative abundances of *Candida* spp. (*C. albicans* and *C. glabrata*) with the exception of one subject specimen containing primarily *Trichosporon asahii*. Our analysis identified these same species and others in additional study visits (Fig. S2). For these subjects, we also compared the bacterial communities (obtained by 16S rRNA gene sequencing and reported in the work of M. Loesche, S. E. Gardner, L. Kalan, J. Horwinski, Q. Zheng, B. P. Hodkinson, A. S. Tyldsley, C. Franciscus, S. L. Hillis, S. Mehta, D. J. Margolis, and E. Grice [submitted for publication]) and discovered in some cases, such as subjects 194 and 198 (visit 0), that fungal identification resulted in the absence of bacterial taxa in the specimen. However, for the majority of wound specimens, mixed fungal-bacterial communities were observed (see Fig. S2 and Fig. S3A in the supplemental material).

### The DFU mycobiome has high interpersonal and intrapersonal variation.

**FIG 2:**
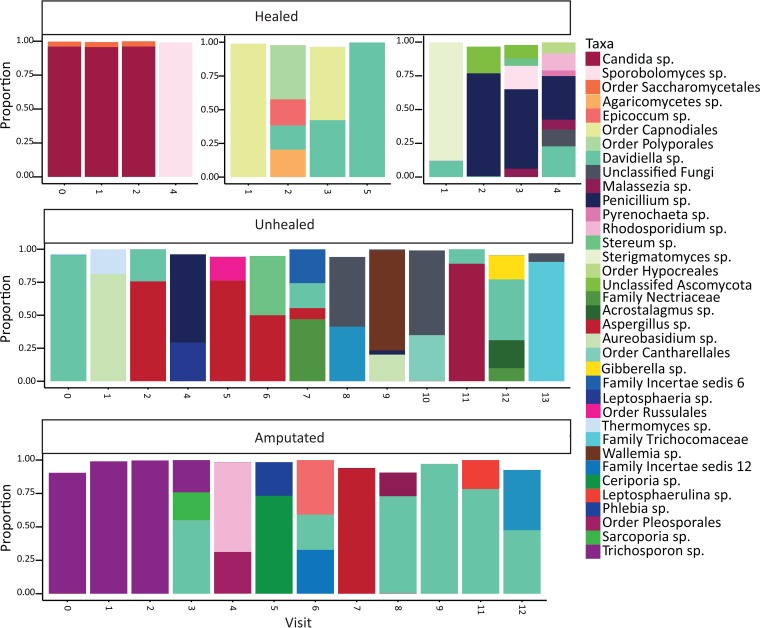
The DFU mycobiome is temporally unstable. Five individual subject timelines showing the relative abundance and structure of fungal communities for DFUs that healed (subjects 145, 117, 159), that did not heal within the 26 weeks of the study (subject 186), or that resulted in an amputation (subject 198). The numbers on the *x* axis are the study visit numbers, and the *y* axis represents proportion of taxa present.

A striking feature of DFU fungal community structures was the absence of a core mycobiome or shared taxa across the study cohort (Fig. 1A). We assessed the temporal stability of fungal communities over time by calculating the weighted UniFrac distances (WUF) between study visits for a single subject. We compared this to mean interpersonal WUF distances at their baseline visit. Mean interpersonal WUF distances were significantly greater than mean intrapersonal WUF distances (Fig. 1D), suggesting that interpersonal variability is greater than intrapersonal variability. However, a high level of dissimilarity occurred in both groups (mean of 0.71 versus 0.67, respectively; *P* < 0.0001 by Wilcoxon rank sum test). At the taxonomic level, community structure was ephemeral, with taxa sometimes appearing or disappearing within one study visit (Fig. 2).

High rates of change between study visits suggest transient colonization or environmental contamination of the wound bed by fungi. Contrary to this hypothesis is the standardized care employed in this study, where a total-contact cast (TCC) (*n* = 87) or a DH boot (*n* = 13) was applied for offloading following wound cleansing and dressing. TCC results in creation of an occlusive environment for the entire foot, minimizing influence from the external environment. We examined the mycobiome community stability of ulcers offloaded with TCC and DH boot but did not observe significant differences between the two offloading methods (data not shown).

### The DFU mycobiome is associated with clinical outcomes.

**FIG 3:**
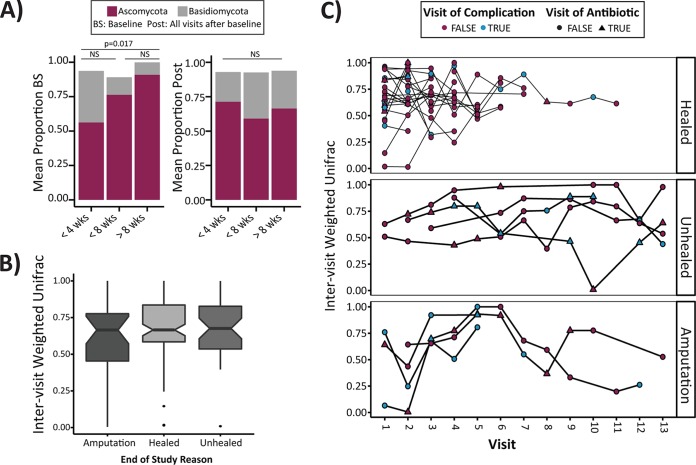
The DFU mycobiome is associated with clinical outcomes. (A) Relative distribution of *Ascomycota* and *Basidiomycota* in specimens grouped by time (in weeks) to heal. Baseline specimens (BS) (left panel) were taken from viable wound tissue prior to sharp debridement and cleansing. All study visits after the baseline study visit are combined (Post) (right panel). *P* values are calculated with an analysis of variance model and *post-hoc* Tukey honest significant difference (HSD) multiple comparison of means. NS, not significant. (B) A boxplot showing intervisit weighted UniFrac distances (*y* axis) by end of study outcome (*x* axis) were not significantly different. Notches display the 95% confidence interval around the median. (C) A timeline of weighted UniFrac distances (*y* axis) plotted by study visit (*x* axis) for individual subjects and grouped by end of study outcome (healed, unhealed after 26 weeks of follow-up, and amputation). Blue dots indicate that a complication was recorded at the study visit. Triangles indicate a study visit at which an antibiotic was administered at or within the previous 2 weeks.

We next determined whether the DFU mycobiome was associated with clinical outcomes. An ideal biomarker would differentiate wound outcomes at the initial presentation of patients; therefore, we analyzed the mycobiomes of the baseline study visit (visit 0) with respect to outcomes, in addition to analyses incorporating all longitudinal data. At the baseline study visit, *Ascomycota* were present in significantly greater relative abundance in wounds that healed in >8 weeks compared to those that healed in <4 weeks (*P* = 0.017 by Tukey *post-hoc* analysis). This distribution was significant only at the initial presentation and *Ascomycota* proportions did not significantly differ between groups after the baseline visit (Fig. 3A). Baseline specimens were taken at the initial clinical presentation and before the wound was surgically debrided of dead tissue and/or biofilms, but the specimens were obtained from viable wound tissue, not necrotic tissue. This suggests that the mycobiome at the presentation visit can be predictive of the time to heal.

We also assessed the stability of the mycobiome and its association with outcomes. Intervisit WUF was not associated with healing outcome; healed or unhealed wounds or wounds on feet that were amputated later display high levels of change over time that were not significantly influenced by a clinical complication or administration of an antibiotic (Fig. 3B and C). Although the mean WUF values were >0.5, indicating high rates of change, in some cases, a trend was observed in subjects that ultimately required an amputation, where the WUF values were lower, meaning mycobiomes from sequential visits were less dissimilar. This suggests a more stable colonization of the fungal community and potentially implicates the formation of a biofilm or infection leading to subsequent chronicity. This is exemplified in the bottom panel of Fig. 2 (subject 198) where the first three study visits of this particular subject were dominated by *Trichosporon asahii*, followed by a shift to a predominant population of *Cladosporidium herbarum* in the later visits. Conversely, the bacterial community stability in this subject is low during the first three visits until the fungal community shifts, and stability for both groups then follows the same general pattern. Similar trends are observed in other subjects and indicate that the stability of the fungal community structure is not independent of the cohabitating bacterial community stability (see Fig. S3B in the supplemental material).

Antibiotics were administered to 31 of 100 subjects at some point during the course of the study. We hypothesized that antibiotic treatment would influence the fungal portion of the microbiome in response to observed bacterial perturbation and disruption (Loesche et al., submitted). In subjects that received antibiotics, the Shannon diversity indices for all visits combined were significantly higher than the indices for the subjects who did not receive an antibiotic (*P* = 0.029 by Wilcoxon rank sum test). However, diversity over time did not significantly fluctuate before, during, or after antibiotic administration. We also examined the class of antibiotic administered and discovered it was nondiscriminating in influencing the overall diversity metrics in these subjects (see Fig. S4 in the supplemental material). In specimens from study visits where a complication was observed, Shannon diversity was also significantly higher (mean Shannon diversity index of 0.98 versus 1.30; *P* < 0.001 by Wilcoxon rank sum test), but visits with complications cooccurred with visits of antibiotic administration only 45% of the time (41/91 total visits).

### Pathogens versus allergens in the DFU mycobiome.

**FIG 4:**
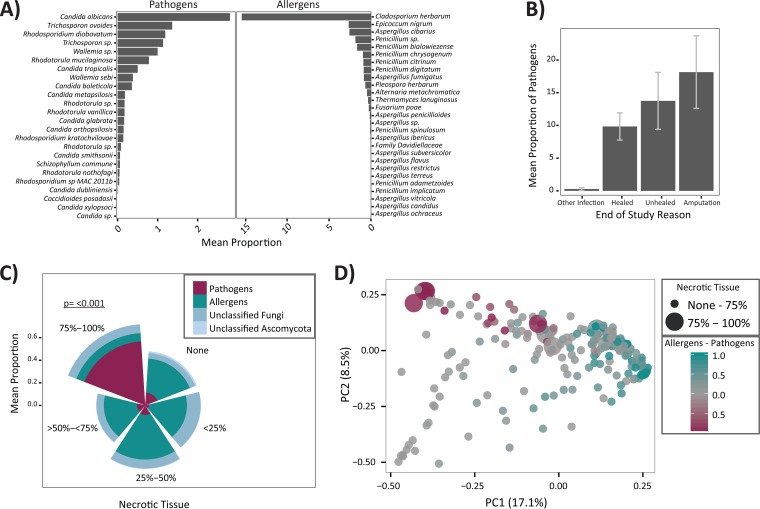
Pathogens are associated with necrotic tissue and poor outcomes. (A) Mean proportion (shown as a percentage) (*x* axis) of pathogen (*y* axis) and allergen (*y* axis) taxa in the data set. (B) Mean proportion of pathogens (*y* axis) by end of study outcome (*x* axis). Error bars indicate standard errors of the means. (C) Mean proportion of pathogens, allergens, unclassified fungi, and unclassified *Ascomycota* in samples grouped by the level of necrotic tissue present in the wound. The level of pathogens is significantly higher in ulcers with >75% necrotic tissue compared to all other levels of necrosis. *P* values are calculated with an analysis of variance model and *post-hoc* Tukey HSD multiple comparison of means. (D) Principal-coordinate plot comparing samples by the weighted UniFrac distance. Percent variation explained by each principal coordinate (PC) is indicated by the percentage next to each axis. Point size indicates necrotic tissue level, with larger points corresponding to greater amounts of necrotic tissue in the wound from which the specimen was taken. The proportions of allergens and pathogens were calculated by subtracting the proportion of pathogens from the proportion of allergens in each sample resulting in a scale of −1 (dominated by pathogens) to +1 (dominated by allergens). A value of zero indicates either zero or equal proportions of each.

We elected to bin taxa into the “pathogens” or “allergens” category, because with respect to skin and cutaneous infection, the majority of taxa identified in our data set fell into one of these two groups of either known/opportunistic skin pathogens or the filamentous fungi often identified as allergenic molds (Fig. 4A). While some members of the “allergens” group, such as *Aspergillus* spp., can also be opportunistic pathogens, this is rare in the context of skin and cutaneous infection so we limited their classification. We first assessed associations between the list of allergen and pathogen phylotypes and the taxa listed in Table 2 using Spearman rank correlations. Taxon correlations were then subjected to hierarchical clustering via hclust. The allergens and pathogens clustered separately, discriminated by their associations with six key taxa (*Aspergillus cibarius*, *Penicillium bialowiezense*, *Epicoccum nigrum*, *Pencillium* sp., *Trichosporon asahii*, and *Candida albicans*). Allergens had positive associations with all six, whereas pathogens did not exhibit any strong associations, positive or negative (see Fig. S5 in the supplemental material).

With respect to clinical outcomes, a trend emerged where the mean proportions of pathogens were higher in nonhealing wounds and those wounds that ultimately resulted in amputation, compared to wounds that healed, though this trend was not statistically significant (Fig. 4B). The mean proportion of allergens was unchanged across all outcome groups (data not shown).

We also examined mean relative abundance of allergens and pathogens with respect to necrosis, because the level of necrotic wound tissue may indicate wound health or deterioration. Strikingly, in wounds with 75 to 100% necrotic tissue, the proportion of allergens is reduced and a highly significant increase in the proportions of pathogens is observed (*P* < 0.001 by analysis of variance and Tukey *post-hoc* analysis) (Fig. 4C). To better visualize this distribution across samples, we constructed an independently calculated weighted UniFrac distance ordination plot overlaid with the relative proportions of allergens and pathogens and the level of necrotic tissue in each sample. Clear separation is observed between the two groups, and pathogens are predominantly found in those samples with high levels of necrosis (Fig. 4D).

Since pathogens may directly contribute to wound necrosis and negative outcomes, the most severe being amputation, we further examined all possible associations between additional clinical factors and the relative abundance of pathogens and allergens and the two dominant taxa in each of those groups (*C. albicans* and *C. herbarum*, respectively). By Spearman rank correlation, allergens were negatively associated with HgbA1c levels (rho = −0.308; *P* = 0.02) and WBC counts (rho = −0.346; *P* = 0.009), suggesting that glucose control and lower levels of inflammation are consistent with allergen colonization (see Fig. S1 in the supplemental material). Specifically, *C. herbarum* was negatively associated with HgbA1c (rho = −0.405; *P* = 0.002) but positively associated with the ulcer surface area (rho = 0.348; *P* = 0.008) and days in the study (rho = 0.279; *P* = 0.038) (Fig. S1). Because the culture-positive samples coincided with wound deterioration and the mean proportion of the group “pathogens” is elevated in nonhealing wounds and necrosis, we looked for associations between both *Candida* spp. and the pathogens group to clinical factors, but no additional significant associations were identified (Fig. S1).

### The DFU mycobiome forms multispecies biofilms with bacteria.

**FIG 5:**
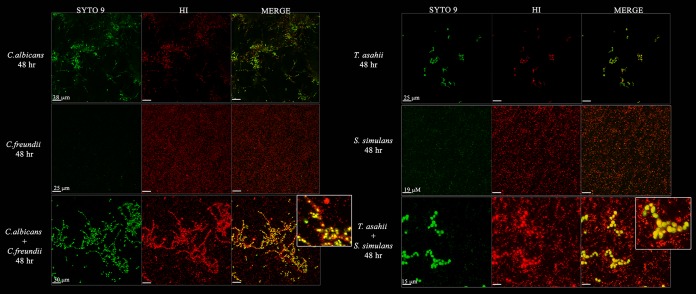
Pathogens form interkingdom biofilms. Fluorescent confocal microscope images of mono- or coculture *Candida albicans* and *Citrobacter freundii* or *Trichosporon asahii* and *Staphylococcus simulans*. Biofilms were grown for 48 h at 37°C on polystyrene plates in RPMI 1640 medium with GlutaMAX supplement, washed to remove planktonic cells, and stained with SYTO 9 and hexidium iodide (HI) prior to imaging. Fungi (large) and bacteria (small) can be distinguished by size. Fungi appear green and bacteria appear red in the merged images. The size of the scale bar for each row is labeled in the first column (SYTO 9) for each culture. The insets are zoomed-in portions of the merged coculture biofilm images.

We hypothesized that biofilm formation occurred in those ulcers where a pathogenic fungal species was detected. Cultures were obtained from samples collected from subjects 145 and 198, representing delayed (>6 weeks) but healed and amputated wounds, respectively (Fig. 2). *C. albicans* was isolated from subject 145, and *T. asahii* was isolated from subject 198. Samples from these subjects also grew the bacterial isolates *Citrobacter freundii* (subject 145) and *Staphylococcus simulans* (subject 198). The ability of each isolate to form a biofilm as monoculture or coculture was assessed and confirmed visually by confocal microscopy. The yeast-bacterium pairs (*C. albicans* plus *C. freundii* and *T. asahii* plus *S. simulans*) readily grew as coculture on agar plates. Biofilm growth was observed for monoculture of each yeast strain with distinct hyphal growth of *C. albicans* and chains or clumping of cells for *T. asahii*. The bacterial strains were also able to grow as biofilms in monoculture, forming distinct microcolonies although in a more dispersed and confluent layer than the yeast monocultures (Fig. 5). Mixed-species biofilms formed within 24 h and further matured over 48 h. The cocultures revealed close interactions between bacterial and yeast cells. The yeast cells appear to form the “core” of the colony, and bacteria associate around the periphery of the cells, coating yeast cells and hyphae as they grow out of the plate to a thickness of approximately 30 µm for *C. albicans* plus *C. freundii* and a thickness of 15 µm for *T. asahii* and *S. simulans* after 48 h (Fig. 5). These observations coupled with quantitative counts of the planktonic and biofilm mono- or cocultures (see Fig. S7 in the supplemental material) suggest that the yeast and bacterial species interact in a noncompetitive manner to form mixed biofilms.

To assess fungal-bacterial interactions with a more global view, we determined Spearman rank correlations between the fungal and bacterial taxa found in >1% abundance in the entire data set. *Corynebacterium* sp. in the order *Actinomycetales* was significantly negatively correlated with *C. albicans* and *Candida parapsilosis*. Members of the *Actinomycetales* are historically rich sources of bioactive small molecules. On the other hand, *C. albicans* was significantly positively correlated with the order *Alcaligenaceae*, a group of Gram-negative *Proteobacteria*.

## DISCUSSION

Chronic nonhealing wounds are host to polymicrobial communities that can form biofilms and interfere with healing processes. Here we utilized high-throughput sequencing of the rRNA internal transcribed spacer ITS1 amplified from DFU specimens collected longitudinally to demonstrate that DFUs contain a diverse repertoire of fungi not recognized clinically or by traditional culture procedures. Our study is the first to identify fungi in a large proportion (80%) of DFUs surveyed. The longitudinal aspect of our study design and standardized care protocol establishes that the mycobiome is highly dynamic and transient in DFUs, with increased fungal diversity in subjects administered antibiotics or experiencing a complication. While the subset of subjects on antibiotics was not large enough to allow for detailed investigation into the short- and long-term effects on the mycobiome, future studies are warranted, incorporating both fungal and bacterial community dynamics in response to antibiotic perturbation.

Studies of the mycobiome in chronic disease are not abundant. It was striking to us that *C. herbarum* was the most abundant species found in ≥1 specimen from 56% of our subjects. Although this saprophytic dematiaceous fungus is widespread in the environment, it has been reported as one of the most common fungal species associated with the human body in different body sites, including the oral, nasal, vaginal, and gut mycobiomes (31, 34, 48–50). Not only are *Cladosporium* spp. sensitizing agents leading to allergic rhinitis, but they are also linked to other human diseases, including an outbreak of fungal meningitis in 2012 (51), and recently, *Cladosporium* spp. were identified in 92 clinical specimens with 28% of those coming from superficial and deep tissues (52). Together, these data indicate that *Cladosporium* spp. should be regarded as a member of the human mycobiome.

*C. albicans* was the second most abundant phylotype identified, present in 47 subjects and 21% of samples. Other *Candida* species identified were *C. parasilopsis* (15%), *C. tropicalis* (9.76%), *C. glabrata* (3.7%), and *C. smithsonii* (2.9%), while *C. boleticola*, *C. dubliniensis*, *C. orthopsilosis*, *C. metapsilosis*, and *C. xylopsoci* were found in less than 3% of the samples. *Candida* abundance can fluctuate during gut microbiome dysbiosis, for instance by antibiotic administration early in life, and has been associated with asthma and allergic airway response to fungal allergens (53–55). Specifically, overgrowth of *C. albicans* in the gut microbiome of mice can provoke sensitization resulting in a CD4^+^-T-cell-mediated response to mold spores that is not observed in mice without microbiota disruption (56). It is not clear whether similar responses occur in other tissues and body sites or whether this response is isolated to the gut-airway axis. The DFU mycobiome is primarily composed of a balance of commensal and pathogenic yeasts (*Candida* spp., *Trichosporon* spp.) and a heterogeneous population of anamorphic fungi recognized as important causes of respiratory allergies (57). Instability in communities both between and within individuals suggests transient or superficial colonization of the DFUs by spores, highlighting an important limitation to sequence-based studies—the inability to determine active metabolism. In this context, however, it is tempting to imagine a scenario by which exposure to fungal spores and their antigens is sufficient to provoke an immunological response that contributes to prolonged inflammation and stalled healing.

Poor perfusion is a hallmark of DFUs and can contribute to impaired healing. Increased fungal diversity in DFUs with reduced oxygenation is consistent with our finding that biofilm-forming yeasts and opportunistic skin commensal pathogens were highly significantly and strongly associated with wound necrosis and poor outcomes. This association was not driven by a single species but a mixed group of pathogens.

This study provides the foundation for further dissection of microbial interactions and their profound influence on disease progression. Here, we demonstrate the ability of yeast-bacterial pairs isolated from DFUs to form mixed biofilms. Two yeast-bacterial pairs were cultured from DFUs identified as having a stable community by our molecular analysis and used to validate fungal-bacterial biofilm formation. To our knowledge, there is little information regarding interactions between *C. albicans* and *C. freundii* or *T. asahii* and *S. simulans*. The results of our *in silico* analysis also suggest that an antagonistic interaction is occurring between *Candida* and *Corynebacterium* species. Continued exploration to determine the magnitude and mechanisms of microbiome interactions in contributing to impaired healing and skin and soft-tissue infection is an important and timely area of research. Observation of diverse fungal communities in chronic nonhealing wounds and their ability to form interkingdom biofilms with both Gram-negative and Gram-positive bacterial species emphasizes the paramount importance but also the complexity of studying whole microbial communities, their interspecies interactions, and implications for chronic disease.

## MATERIALS AND METHODS

### Study design.

From September 2008 through October 2012, 100 subjects were enrolled in a prospective cohort to sample the DFU microbiota and measure outcomes. Subjects were recruited through local-media advertisements and from outpatient clinics at the University of Iowa Hospitals and Clinics (UIHC) and the Iowa City Veteran’s Affairs Medical Center (VA). Samples for microbiota analyses were collected at the initial presentation (visit 0 [V0]) and every 2 weeks until the following: (i) the DFU healed, (ii) the foot was amputated, or (iii) 26 weeks of follow-up elapsed (visit 1 [V1] to V12). The Institutional Review Boards (IRBs) at the University of Iowa (IRB number 200706724) and the University of Pennsylvania approved the study procedures (IRB number 815195). Informed consent was obtained from all participants in writing.

Wound management was standardized to aggressive sharp debridement of necrotic tissue in the wound bed at baseline and wound edge callus removal every 2 weeks followed by nonantimicrobial dressing application (i.e., Lyofoam; Molnlycke Health Care). Ulcers were offloaded with total-contact casts (87 subjects) or DH boots (13 subjects). Topical antimicrobial or system antibiotic administration was not included unless an infection-related complication was present at the beginning of the study (baseline) or occurred within the study period. Data were collected at baseline and longitudinally every 2 weeks until the wound healed or 26 weeks had elapsed.

### Study variables. (i) Clinical factors.

Demographic variables were collected at the initial or baseline visit, including age, sex, diabetes type and duration, and duration of the study ulcer using subject self-report and medical records. At each study visit, glycemic control was measured by the levels of hemoglobin A1c and blood glucose. Inflammatory (erythrocyte sedimentation rate [ESR] and C-reactive protein) and immune (white blood cell counts) markers were determined with standard laboratory tests. Each subject was also assessed for ischemia using the ankle-brachial and toe-brachial index and for neuropathy using the 5.07 Semmes-Weinstein monofilament test. Transcutaneous oxygen pressure was measured at baseline and at each follow-up visit, using a transcutaneous oxygen monitor (Novametrix840; Novametrix Medical Systems Inc.). Ulcer location was categorized as forefoot, midfoot, or hind foot. The level of necrotic tissue was defined as black, gray, or yellow tissue in the wound bed measured using a Likert scale as the percentage of the total wound area binned according to 0 to 25%, 25 to 50%, 50 to 75%, or 75 to 100% necrotic tissue.

### (ii) Outcomes.

Healing and infection-related complications were assessed every 2 weeks. Ulcer closure was determined using the Wound Healing Society’s definition of “an acceptably healed wound,” a valid and reliable definition (58). “Development of infection-related complications” was defined as wound deterioration, new osteomyelitis, and/or amputations due to DFU infections.

Wound deterioration was defined as the development of frank erythema and heat and an increase in size of >50% over baseline. Two members of the research team independently assessed each DFU for erythema and heat. Two members of the research team independently assessed size using the VeVMD digital software system (Vista Medical, Winnipeg, Manitoba, Canada) and procedures previously described (59). A cotton-tipped swab, placed in the deepest aspect of the DFU, was marked where the swab intersected with the plane of the periwound skin. The distance between the tip of the swab and the mark was measured as ulcer depth using a centimeter ruler.

Osteomyelitis was assessed using radiographs and magnetic resonance imaging (MRI) at baseline and during follow-up visits when subjects presented with new tracts to bone, wound deterioration, elevated temperature, elevated white blood cell count, elevated erythrocyte sedimentation rate, or elevated C-reactive protein. If these indicators were absent at follow-up, radiographs were not retaken. Subjects experiencing new amputations had their medical records reviewed by the research team to ensure that amputations were due to DFU infection and not some other reason.

### (iii) Sequencing of fungal ITS1 rRNA region.

Ulcer specimens were collected using the Levine technique and established protocols (30). DNA was isolated from swab specimens as previously described (21). The ITS1F (F stands for forward) (CTTGGTCATTTAGAGGAAGTAA) and ITS2R (R stands for reverse) (GCTGCGTTCTTCATCGATGC) primers were used for PCR amplification, each having a linker sequence, a sample-specific GoLay12 index, and an Illumina adapter to amplify the ITS1 region of the fungal rRNA region. These indexed primers were used in combinations that made it possible to multiplex up to 576 (24 × 24) samples at a time. Each sample (along with one mock community, three buffer controls, and two water controls) was amplified in duplicate, combined, and cleaned using the Agencourt AMPure XP bead-based PCR purification system (Beckman Coulter). PCR mixtures contained 9.65 µl PCR-clean water, 1.25 µl 10× Accuprime buffer II (Invitrogen), 0.1 µl Accuprime high-fidelity *Taq* (Invitrogen), 0.25 µl each of the forward and reverse primers (at 10 µM concentration), and 1.0 µl genomic DNA. Reaction mixtures were held at 94°C for 3 min to denature the DNA, with amplification proceeding for 35 cycles, with 1 cycle consisting of 94°C for 45 s, 56°C for 60 s, and 72°C for 90 s; a final extension of 10 min at 72°C was performed. Purified amplicon pools were quantified using the Quant-IT double-stranded DNA (dsDNA) high-sensitivity assay kit (Invitrogen) and a microplate reader (ThermoFisher Scientific). A composite sample for sequencing was made by combining equimolar ratios of amplicons from the samples, followed by gel purification with a Qiagen MinElute gel extraction kit to remove potential contaminants and PCR artifacts (the acceptable size window for amplicons was 200 to 1,000 bp in length). The pooled DNA was quantified using a Qubit fluorometer (Life Technologies), and φX174 genomic DNA was spiked into the sample at ~40% prior to sequencing. MiSeq 300- bp paired-end “V3” sequencing was performed. Additional negative controls were processed as described above, except the ITS1F and ITS2R primers were used without bar-coded adapters and amplicons were sequenced by standard Sanger sequencing.

The MiSeq ITS libraries were preprocessed using an in-house pipeline that includes read quality control (QC), barcode demultiplexing, paired-end assembly, and linker cleaning steps. The pipeline procedures are briefly explained below.

1. Read QC. Raw read quality was checked for the average and range of the Phred quality scores along the reads (1 to 300 bp) for both forward and reverse reads independently.

2. Read demultiplexing. Our MiSeq ITS library construction protocol utilizes a customized barcode system with both forward and reverse bar codes embedded near the 5′ termini of both reads; thus, the forward and reverse bar codes are first spliced and concatenated from the corresponding reads, and then read pairs are demultiplexed using the Flexbar program (v2.4) (60) with default settings.

3. Paired-end assembly. Demultiplexed paired-end reads are assembled (merged) using the PEAR (v0.9.0) program (61) with default settings.

4. Bar code/linker cleaning. Our customized bar code/primer system incorporates a linker region between the actual bar codes and PCR primers to increase the heterogeneity of the amplicons for successful Illumina sequencing. These in-line bar code and linker sequences are cleaned from final assembled amplicons by in-house Perl scripts, in a way that is based only on the length of the bar codes/linkers, which guarantees a successful removal.

The PIPITS pipeline was used for ITS1 processing (45). Briefly, the ITS1 region was extracted with ITSx (46), clustered into operational taxonomic units (OTUs) with VSEARCH (https://github.com/torognes/vsearch) at 97% sequence similarity and chimera removal performed using the UNITE UCHIME reference data set. Representative sequences were assigned taxonomic classification with the RDP classifier against the UNITE fungal ITS reference data set (62) at a confidence threshold of 0.85. Contaminants found in negative controls (corresponding to the taxon *Saccharomyces cerevisiae* or *Alternaria eichornia*) were removed at the OTU level (4 OTUs removed) followed by subsampling 500 sequences per sample. The Shannon diversity index, Simpson diversity index (1-Dominance), Faith’s phylogenetic distance (PD), and number of observed species (richness) were calculated using the QIIME 1.8.0 alpha_diversity.py script (63). Beta-diversity metrics were calculated with the QIIME 1.8.0 beta_diversity.py script.

### Fungal and bacterial manipulation. (i) Isolation.

Yeast and bacterial isolates were grown from wound swabs collected in Trypticase soy broth (TSB). Briefly, 100 µl of TSB containing the swab was plated onto yeast-mold agar (YM agar; Neogen, Lansing, MI) and incubated at 25°C for up to 7 days. Individual colonies were picked and grown on YM or TSB agar plates to be made into glycerol stocks for long-term storage. All strains isolated were identified by amplification of the ITS1 region (primers described above without adapters) or 16S rRNA gene (16S 27’F and 534’R) and Sanger sequencing. Secondary confirmation was obtained by matrix-assisted laser desorption ionization−time of flight (MALDI-TOF) mass spectrometry at the Pennsylvania Animal Diagnostic Laboratory System.

The yeast isolate grown from subject 198 was identified as *Trichosporon asahii* by Sanger sequencing and a BLAST search against the NCBI nucleotide (nt) database and UNITE database. The sequence was compared to the OTU identified as *Trichosporon ovoides* as part of the PIPITS pipeline and found to have 100% identity, so the OTU was reclassified as *T. asahii*.

### (ii) Biofilm growth.

Isolates were grown overnight at 37°C on YM agar (fungi) or Trypticase soy agar (TSA) (bacteria). Colonies were scraped into 0.89% NaCl and diluted to an optical density at 600 nm (OD_600_) of 0.08 to 0.1 with the exception of *T. asahii* (OD_600_ of 0.17 to 0.2). Bacterial suspensions were diluted 1/10 into RPMI 1640 medium with GlutaMAX supplement (ThermoFisher Scientific, Waltham, MA). The inoculums were then added in a 1/10 dilution to a final volume of 4 ml RPMI 1640 medium in 35-mm polystyrene plates. Cultures were incubated stationary at 37°C for 24 or 48 h to allow adhesion and growth. The medium was removed, and the biofilms were washed twice with 1 ml of sterile 0.89% NaCl to remove nonadherent cells. The biofilms were stained with the LIVE BacLight bacterial Gram stain kit (ThermoFisher Scientific, Waltham, MA) with SYTO 9 (480 nm/500 nm) and hexidium iodide (480 nm/625 nm) as a 2-ml solution in water and according to the kit instructions. The stain was removed, and deionized water was added to the biofilms prior to imaging on a Leica TCS SP5 microscope with 20× objective. Images were postprocessed with Volocity software (PerkinElmer, Waltham, MA). The maximum projection for each image was used to generate Fig. 5.

Quantitative counts of mono- and cocultures were performed by serial dilution of planktonic cells (media), wash media (washed twice with 1 ml), and 0.89% saline containing adherent cells that were scraped and resuspended (*n* = 2). The dilutions were plated onto nonselective media (YM or TSA) and incubated for 16 to 18 h at 37°C. Colonies were counted, and the total CFU were calculated.

### Data analyses.

The R statistical package (64) was used for all computations unless described elsewhere. The classification of “pathogens” or “allergens” was performed manually based on classification in literature. Statistical methods are described in the text and figure legends. Correlations between microbiome and clinical features were determined by calculating the Spearman coefficient.

### Accession numbers.

All sequence data are publicly available on the NCBI Sequence Read Archive with accession number SRP076355 and BioProject accession number PRJNA324668.

## SUPPLEMENTAL MATERIAL

Figure S1  Heatmap illustrating positive (yellow) and negative (purple) correlations between microbiome factors and clinical factors at baseline. Correlations were calculated by the Spearman correlation coefficient. Significant correlations are marked with an asterisk (*P* ≤ 0.05), and rho and *P* values are summarized in Table S1 in the supplemental material. Download Figure S1, PDF file, 0.5 MB

Figure S2  Subjects with positive yeast culture result. The study visit that yielded a culture-positive result is marked by an asterisk, and the species identified are given. Relative abundance plots are shown for taxa identified by ITS1 analysis and found in >5% abundance in each sample. Numbers on the *x* axis represent the study visit number, and the *y* axis shows the proportion of taxa. Download Figure S2, PDF file, 2 MB

Figure S3  Subjects with positive yeast culture result. (A) Relative abundance plots are shown for bacterial taxa identified by 16S rRNA gene analysis and found in >1% abundance in the entire data set (384 samples). Numbers on the *x* axis represent the study visit number, and the *y* axis shows the proportion of taxa. (B) A timeline of weighted UniFrac distances (WUF) (*y* axis) plotted by study visit (*x* axis) for individual subjects. Blue lines indicate the fungal WUF distances, and the red lines indicate the bacterial WUF distances over time for each subject. Download Figure S3, PDF file, 0.6 MB

Figure S4  Shannon diversity indices for subjects who were given an antibiotic (*n* = 31) during the course of the study or who experienced a complication (*n* = 30). (A) Shannon diversity indices for all subjects who received an antibiotic at least once during the study period or not at all. (B) Shannon indices for samples obtained before, during, or after antibiotic administration. (C) Shannon indices for subjects at the visit that a complication was observed or no complication. (D) Shannon indices for samples corresponding to different antibiotic classes. Adjusted (Holm) *P* values were calculated by pairwise Wilcoxon rank sum test. Misc, miscellaneous. Download Figure S4, PDF file, 0.6 MB

Figure S5  Dendrogram and heatmap illustrating positive (blue) and negative (red) correlations between the taxa found in >1% abundance across the entire sample set and the pathogen and allergen groups. Correlations were calculated by the Spearman correlation coefficient. Allergen and pathogen groups are indicated with pink and blue bars, respectively, at the top of the heatmap. Download Figure S5, PDF file, 0.8 MB

Figure S6  Dendrogram and heatmap illustrating positive (pink) and negative (blue) correlations between the fungal and bacterial taxa found in >1% abundance across the entire sample set. Correlations were calculated by the Spearman correlation coefficient. Significant correlations are marked with an asterisk (*P* < 0.05). Download Figure S6, PDF file, 0.7 MB

Figure S7  Quantitative culture data. (A) Quantitative counts for *C. albicans* and *C. freundii* planktonic and biofilm populations growing as monoculture (m) or coculture (cc). (B) Quantitative counts for *T. asahii* and *S. simulans* planktonic and biofilm populations growing as monoculture (m) or coculture (cc). All biofilm counts were obtained after washing the biofilms twice with 1 ml of sterile water. Counts are averaged across a minimum of two replicates and two serial dilutions per replicate. Download Figure S7, PDF file, 0.2 MB

Table S1  Summary of Spearman correlation coefficients and *P* values for Fig. S1 in the supplemental material. Table S1, PDF file, 0.1 MB

Table S2  Differentially abundant taxa between forefoot and hind foot wounds (*P* values calculated by analysis of variance and adjusted by the Benjamini and Hochberg method). Table S2, PDF file, 0.1 MB
