# Supplementary figures and images for: Redefining the Chronic-Wound Microbiome: Fungal Communities Are Prevalent, Dynamic, and Associated with Delayed Healing
Source: mBio. 2016 Sep 6;7(5):e01058-16. doi: 10.1128/mBio.01058-16 (PMC5013295; doi:10.1128/mBio.01058-16)

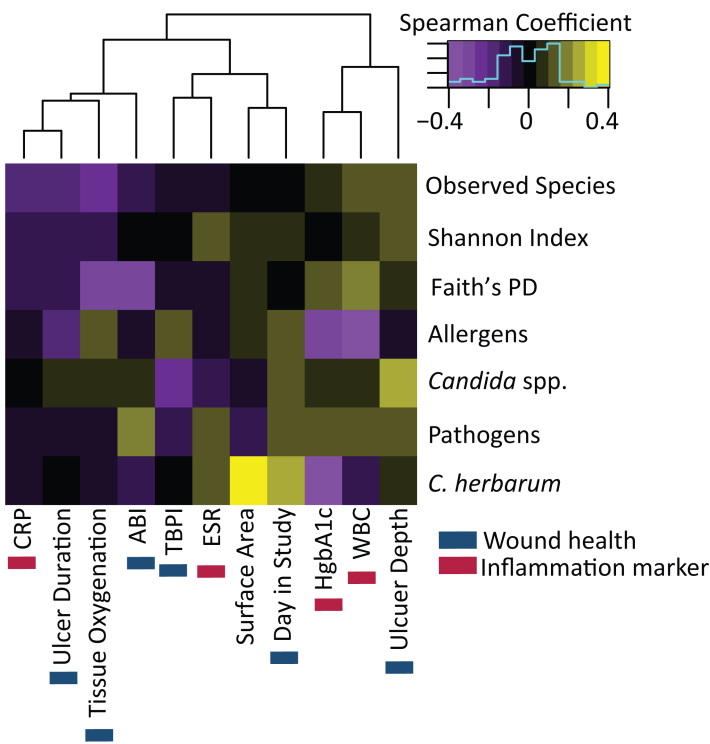

Supplement: Figure S1 — Heatmap illustrating positive (yellow) and negative (purple) correlations between microbiome factors and clinical factors at baseline. Correlations were calculated by the Spearman correlation coefficient. Significant correlations are marked with an asterisk (P ≤ 0.05), and rho and P values are summarized in Table S1 in the supplemental material. Download [file mbo004162957sf1.pdf]

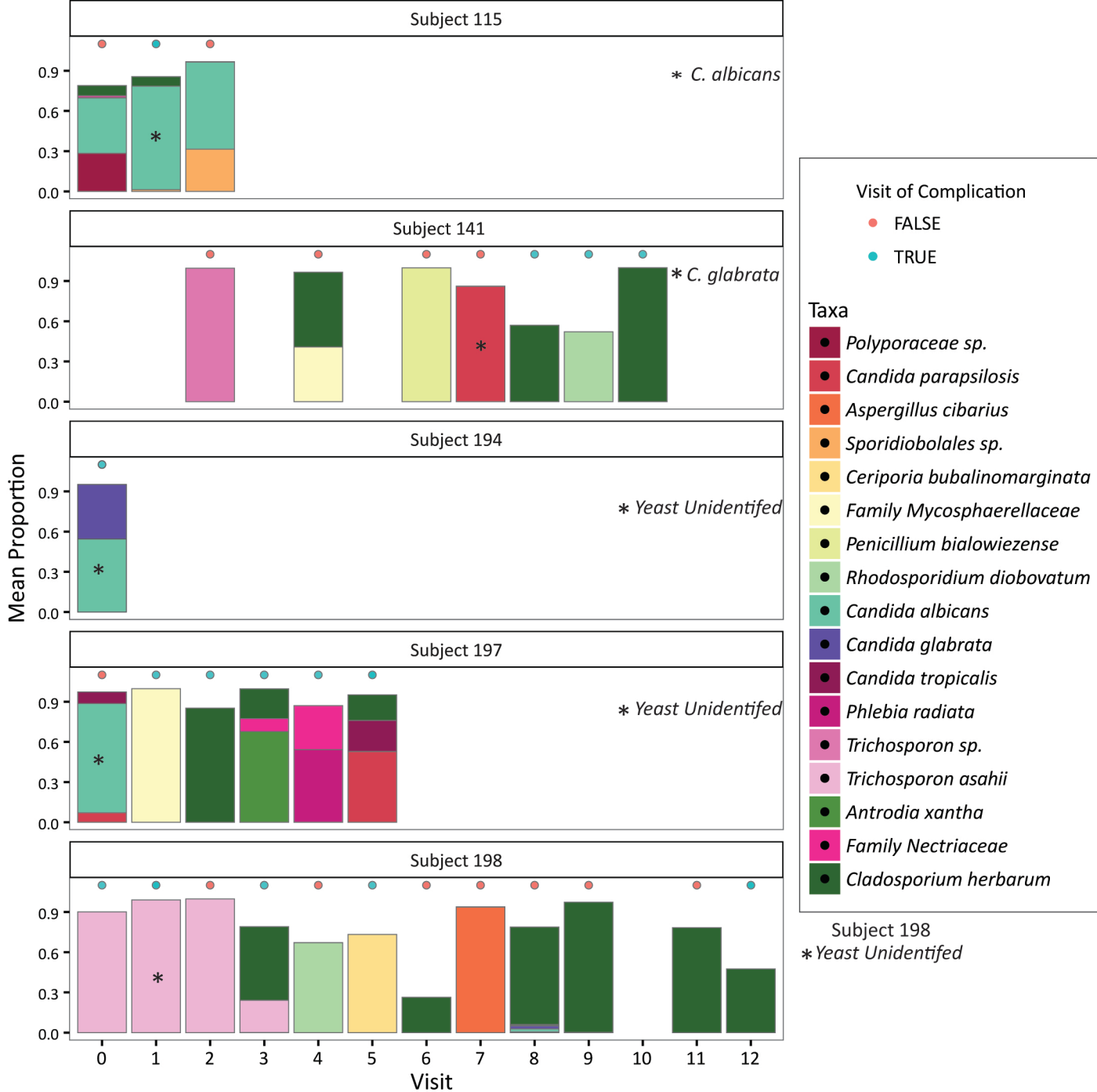

Supplement: Figure S2 — Subjects with positive yeast culture result. The study visit that yielded a culture-positive result is marked by an asterisk, and the species identified are given. Relative abundance plots are shown for taxa identified by ITS1 analysis and found in >5% abundance in each sample. Numbers on the x axis represent the study visit number, and the y axis shows the proportion of taxa. Download [file mbo004162957sf2.pdf]

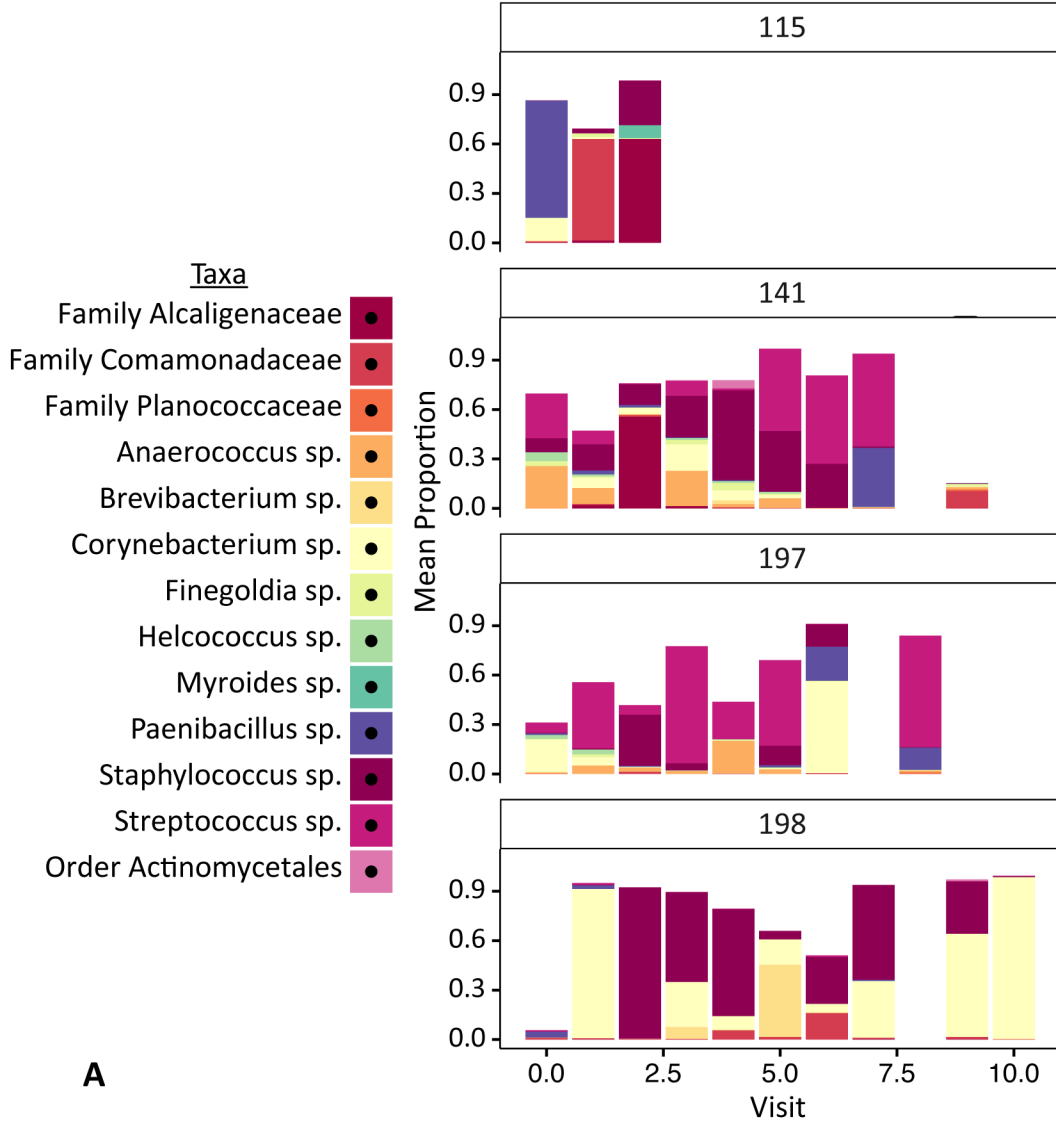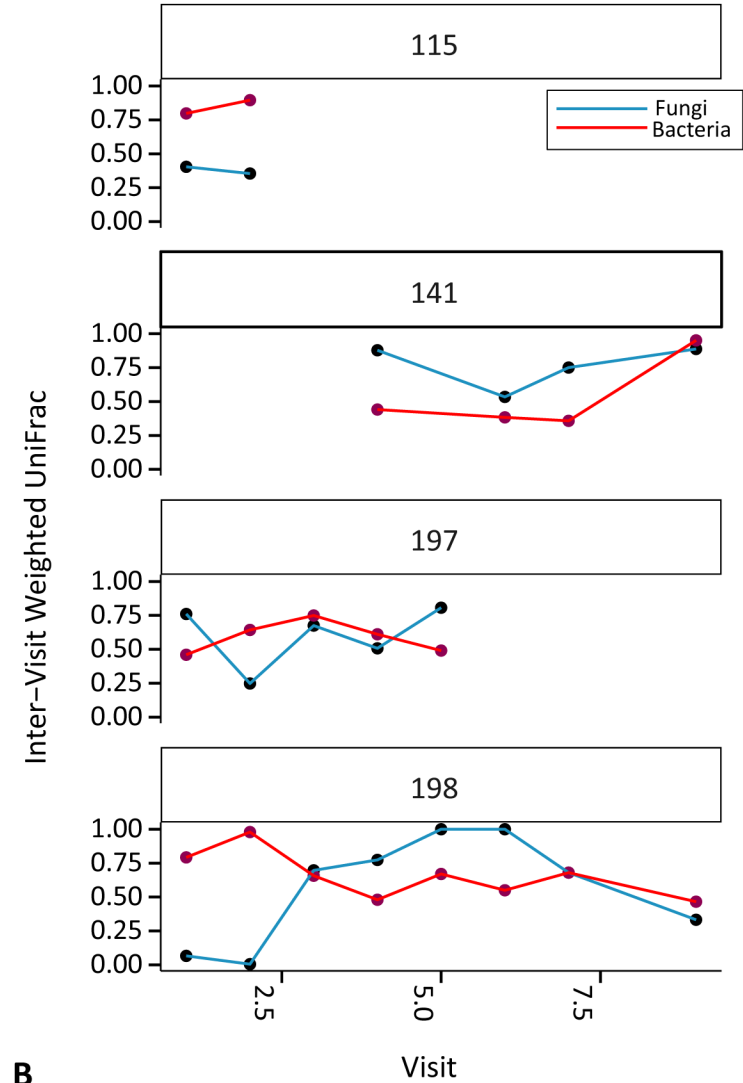

Supplement: Figure S3 — Subjects with positive yeast culture result. (A) Relative abundance plots are shown for bacterial taxa identified by 16S rRNA gene analysis and found in >1% abundance in the entire data set (384 samples). Numbers on the x axis represent the study visit number, and the y axis shows the proportion of taxa. (B) A timeline of weighted UniFrac distances (WUF) (y axis) plotted by study visit (x axis) for individual subjects. Blue lines indicate the fungal WUF distances, and the red lines indicate the bacterial WUF distances over time for each subject. Download [file mbo004162957sf3.pdf]

A)

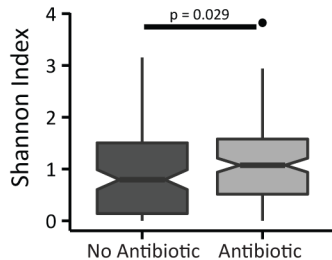

B)

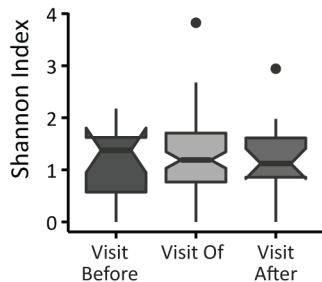

C)

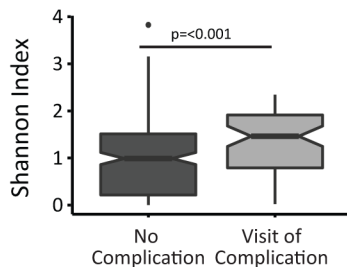

D)

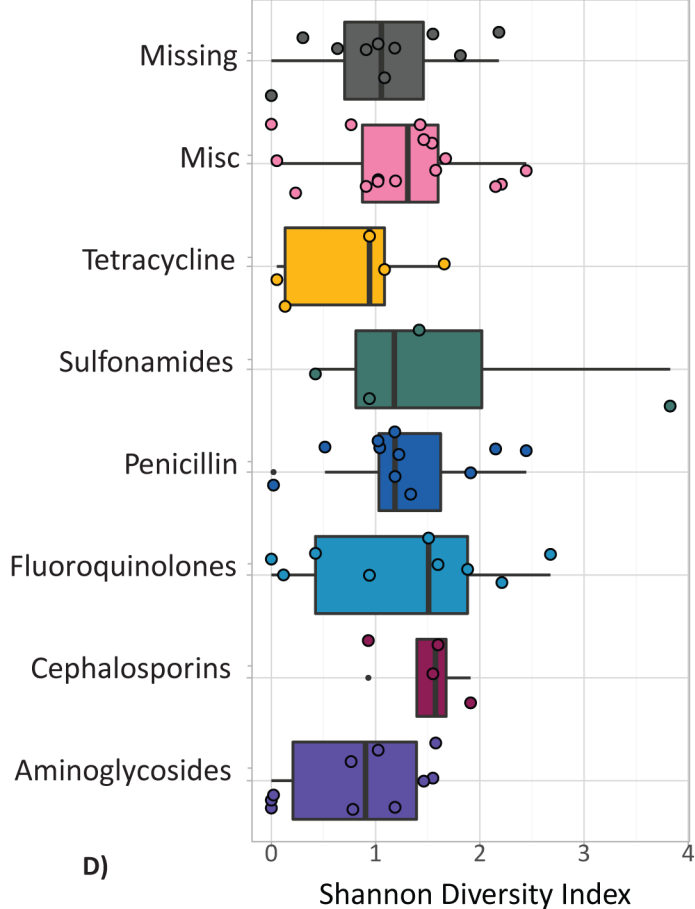

Supplement: Figure S4 — Shannon diversity indices for subjects who were given an antibiotic (n = 31) during the course of the study or who experienced a complication (n = 30). (A) Shannon diversity indices for all subjects who received an antibiotic at least once during the study period or not at all. (B) Shannon indices for samples obtained before, during, or after antibiotic administration. (C) Shannon indices for subjects at the visit that a complication was observed or no complication. (D) Shannon indices for samples corresponding to different antibiotic classes. Adjusted (Holm) P values were calculated by pairwise Wilcoxon rank sum test. Misc, miscellaneous. Download [file mbo004162957sf4.pdf]

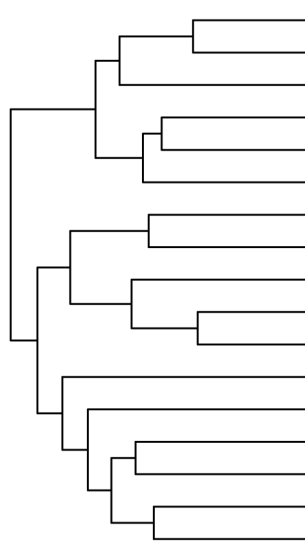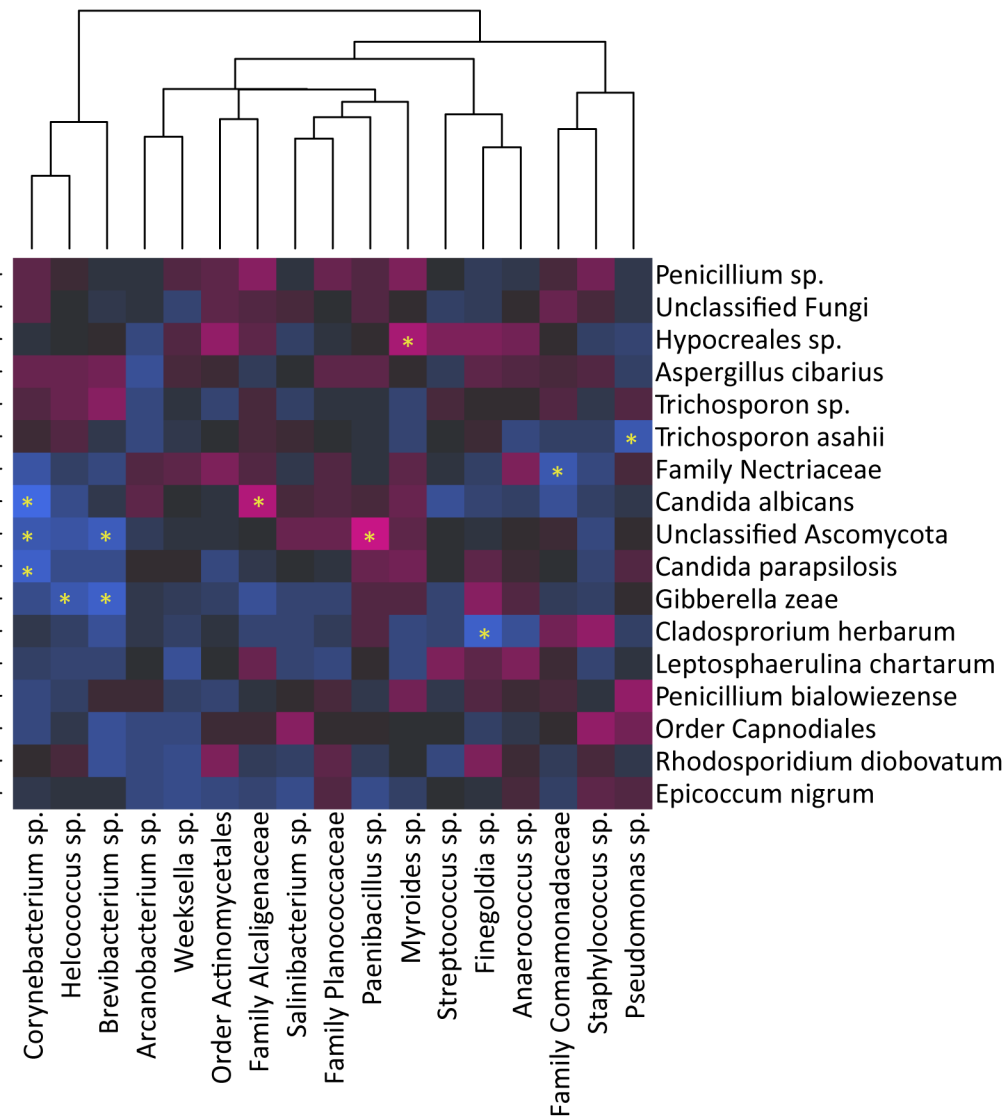

Supplement: Figure S6 — Dendrogram and heatmap illustrating positive (pink) and negative (blue) correlations between the fungal and bacterial taxa found in >1% abundance across the entire sample set. Correlations were calculated by the Spearman correlation coefficient. Significant correlations are marked with an asterisk (P < 0.05). Download [file mbo004162957sf6.pdf]

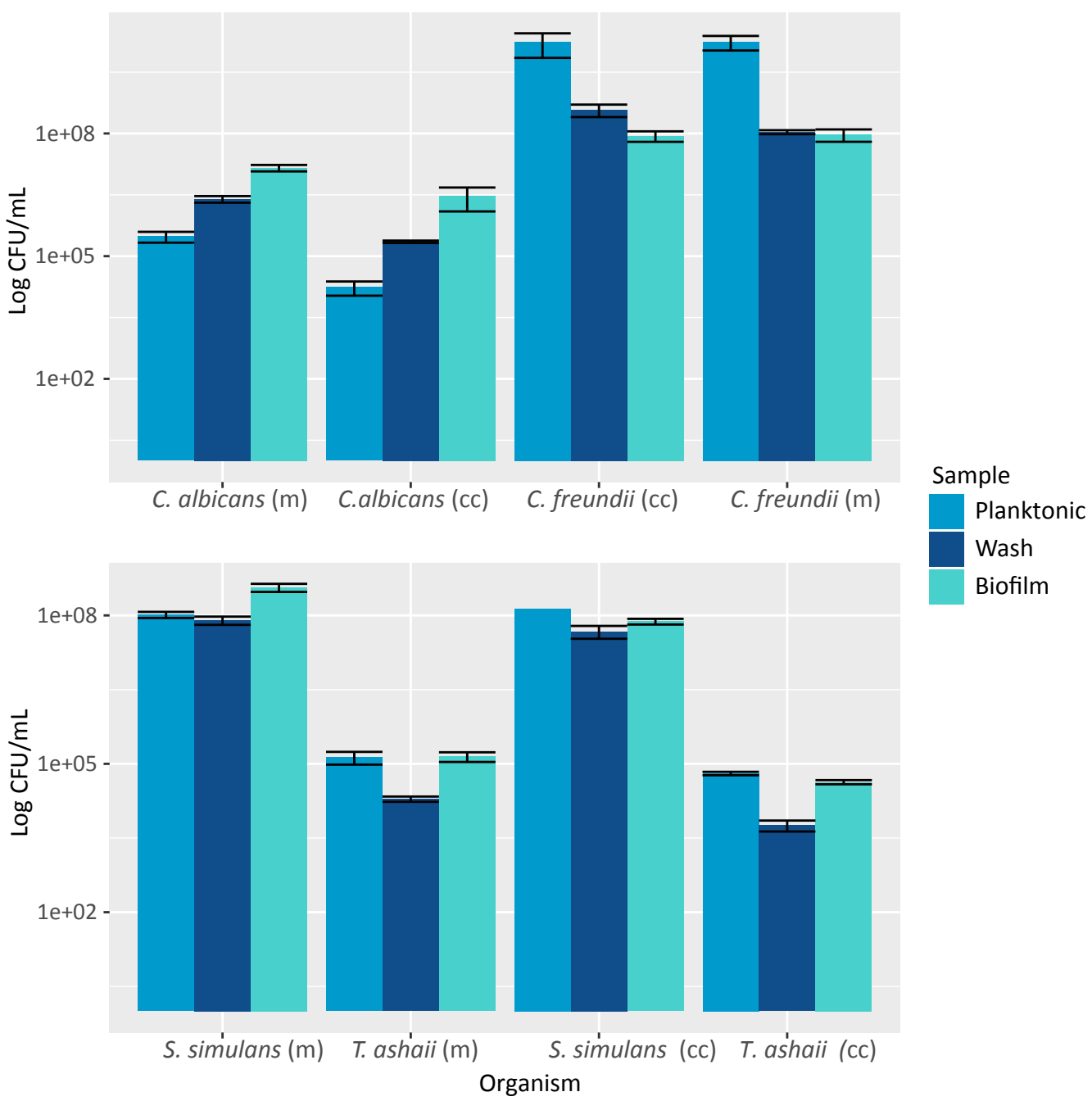

Supplement: Figure S7 — Quantitative culture data. (A) Quantitative counts for C. albicans and C. freundii planktonic and biofilm populations growing as monoculture (m) or coculture (cc). (B) Quantitative counts for T. asahii and S. simulans planktonic and biofilm populations growing as monoculture (m) or coculture (cc). All biofilm counts were obtained after washing the biofilms twice with 1 ml of sterile water. Counts are averaged across a minimum of two replicates and two serial dilutions per replicate. Download [file mbo004162957sf7.pdf]
