# Supplementary material for: Redefining the Chronic-Wound Microbiome: Fungal Communities Are Prevalent, Dynamic, and Associated with Delayed Healing
Source: mBio. 2016 Sep 6;7(5):e01058-16. doi: 10.1128/mBio.01058-16 (PMC5013295; doi:10.1128/mBio.01058-16)
Supplement: Figure S5 — Dendrogram and heatmap illustrating positive (blue) and negative (red) correlations between the taxa found in >1% abundance across the entire sample set and the pathogen and allergen groups. Correlations were calculated by the Spearman correlation coefficient. Allergen and pathogen groups are indicated with pink and blue bars, respectively, at the top of the heatmap. Download [file mbo004162957sf5.pdf]

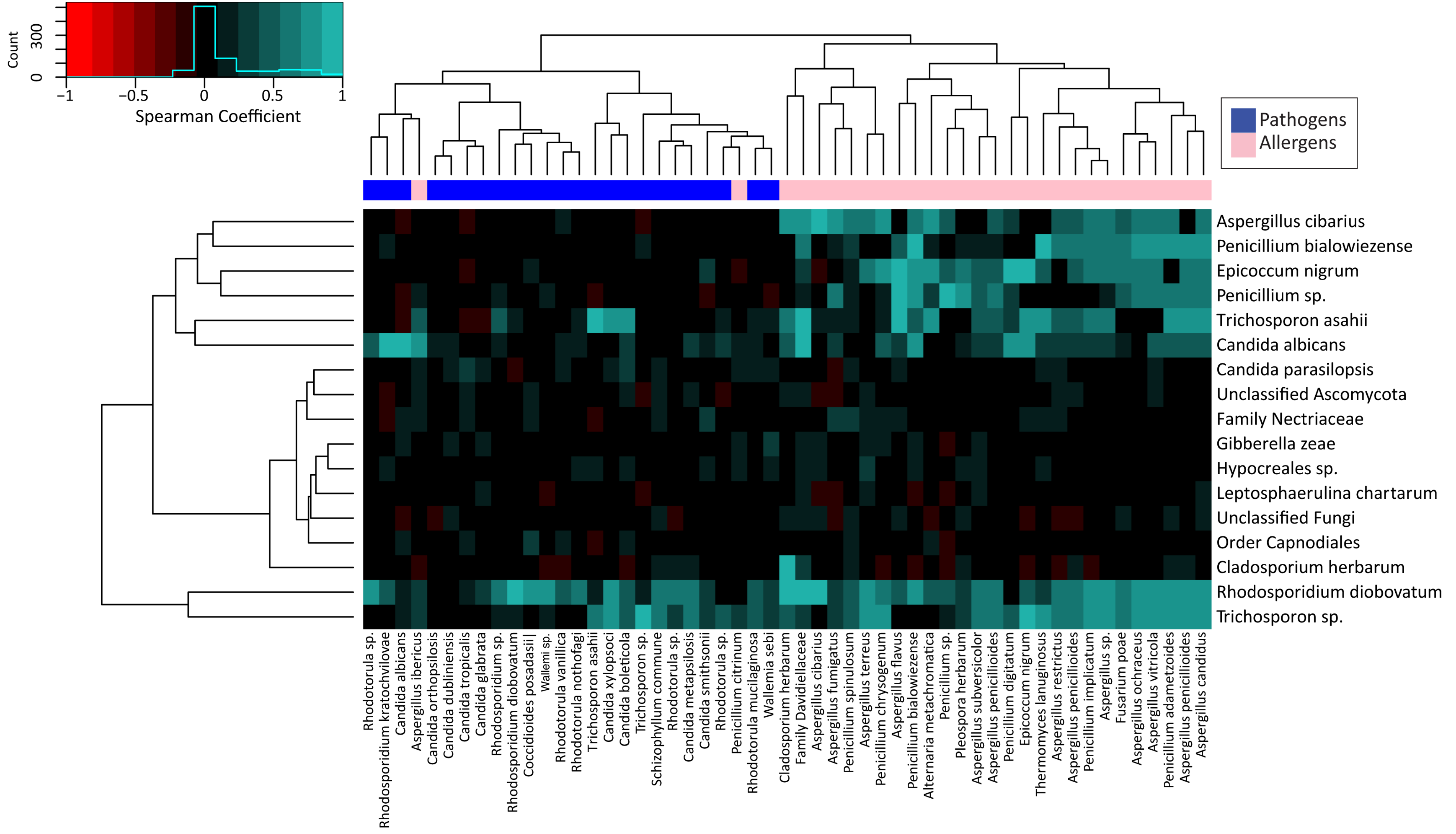

Count
0
300
−1
−0.5
0
0.5
1
Spearman Coefficient
Pathogens
Allergens
Aspergillus cibarius
Penicillium bialowiezense
Epicoccum nigrum
Penicillium sp.
Trichosporon asahii
Candida albicans
Candida parasilopsis
Unclassified Ascomycota
Family Nectriaceae
Gibberella zeae
Hypocreales sp.
Leptosphaerulina chartarum
Unclassified Fungi
Order Capnodiales
Cladosporium herbarum
Rhodosporidium diobovatum
Trichosporon sp.
Rhodotorula sp.
Rhodosporidium kratochvilovae
Candida albicans
Aspergillus ibericus
Candida orthopsilosis
Candida dubliniensis
Candida tropicalis
Candida glabrata
Rhodosporidium sp.
Rhodosporidium diobovatum
Coccidioides posadasii
Wallemi sp.
Rhodotorula vanillica
Rhodotorula nothofagi
Trichosporon asahii
Candida xylopsoci
Candida boleticola
Trichosporon sp.
Schizophyllum commune
Rhodotorula sp.
Candida metapsilosis
Candida smithsonii
Rhodotorula sp.
Penicillium citrinum
Rhodotorula mucilaginosa
Wallemia sebi
Cladosporium herbarum
Family Davidiellaceae
Aspergillus cibarius
Aspergillus fumigatus
Penicillium spinulosum
Aspergillus terreus
Penicillium chrysogenum
Aspergillus flavus
Penicillium bialowiezense
Alternaria metachromatica
Penicillium sp.
Pleospora herbarum
Aspergillus subversicolor
Aspergillus penicillioides
Penicillium digitatum
Epicoccum nigrum
Thermomyces lanuginosus
Aspergillus restrictus
Aspergillus penicillioides
Penicillium implicatum
Aspergillus sp.
Fusarium poae
Aspergillus ochraceus
Aspergillus vitricola
Penicillium adametzoides
Aspergillus penicillioides
Aspergillus candidus
