# Supplementary material for: Redefining the Chronic-Wound Microbiome: Fungal Communities Are Prevalent, Dynamic, and Associated with Delayed Healing
Source: mBio. 2016 Sep 6;7(5):e01058-16. doi: 10.1128/mBio.01058-16 (PMC5013295; doi:10.1128/mBio.01058-16)
Supplement: Table S1 — Summary of Spearman correlation coefficients and P values for Fig. S1 in the supplemental material. [file mbo004162957st1.pdf]

Table S1: Summary of Spearman correlation coefficients and p-values for supplementary figure 1.

	HgbA1c		Tissue oxygenation		Ulcer depth		Ulcer duration		Ulcer surface area		WBC	
	rho	p-value	rho	p-value	rho	p-value	rho	p-value	rho	p-value	rho	p-value
Observed species	0.0915	0.4793	-0.2582	**0.0464**	0.1082	0.4026	-0.1843	0.1516	-0.0126	0.9224	0.1013	0.4373
Shannon index	0.0175	0.8923	-0.1284	0.3281	0.1197	0.354	-0.1	0.4394	0.0903	0.4853	0.0521	0.6901
Faith's PD	0.1218	0.3456	-0.2953	**0.022**	0.0415	0.7489	-0.1436	0.2655	0.0649	0.616	0.2094	0.1052
Allergens	-0.308	**0.0198**	0.1371	0.3182	-0.0436	0.7472	-0.1639	0.2233	0.0779	0.5646	-0.3463	**0.0089**
Candida sp.	0.0402	0.7666	0.0465	0.7358	0.2255	0.0917	0.0899	0.506	-0.0387	0.7752	0.0497	0.716
Pathogens	0.0953	0.4808	-0.0781	0.571	0.1517	0.26	-0.0555	0.6818	-0.1281	0.3422	0.1449	0.2866
C. herbarum	-0.4053	**0.0018**	-0.0531	0.7002	0.0346	0.7984	0.0063	0.9631	0.3482	**0.0079**	-0.1452	0.2857

	CRP		TBPI		ABI		Days in study		ESR	
	rho	p-value	rho	p-value	rho	p-value	rho	p-value	rho	p-value
Observed species	-0.1615	0.2099	-0.0453	0.7813	-0.1322	0.5678	0.0185	0.8891	-0.0506	0.6985
Shannon index	-0.1117	0.3874	-0.0305	0.852	-0.008	0.9725	0.0319	0.8103	0.1291	0.3212
Faith's PD	-0.1074	0.4061	-0.0926	0.5697	-0.3254	0.15	-0.0243	0.8552	-0.0705	0.5892
Allergens	-0.0716	0.5965	0.1553	0.3588	-0.05	0.839	0.1096	0.4257	-0.0401	0.7694
Candida sp.	-0.0299	0.8252	-0.2354	0.1608	0.087	0.7231	0.1025	0.4563	-0.113	0.4072
Pathogens	-0.0934	0.4894	-0.1055	0.5342	0.182	0.4559	0.1124	0.4138	0.0956	0.4836
C. herbarum	-0.0361	0.7899	-0.0275	0.8716	-0.1344	0.5832	0.2794	**0.0388**	0.1121	0.4109
