# Supplementary material for: Redefining the Chronic-Wound Microbiome: Fungal Communities Are Prevalent, Dynamic, and Associated with Delayed Healing
Source: mBio. 2016 Sep 6;7(5):e01058-16. doi: 10.1128/mBio.01058-16 (PMC5013295; doi:10.1128/mBio.01058-16)
Supplement: Table S2 — Differentially abundant taxa between forefoot and hind foot wounds (P values calculated by analysis of variance and adjusted by the Benjamini and Hochberg method). [file mbo004162957st2.pdf]

Table S2: Differentially abundant taxa between forefoot and hindfoot wounds (p-values calculated by analysis of variance and adjusted by the Benjamini & Hochberg method)

Taxa	p-value
Order Saccharomycetales	0.024
Mycosphaerellaceae sp.	0.492
Candida albicans	2.19 E-05
Tremellales sp.	0.0191
Sporobolomyces ruberrimus	0.0191
Phlebia radiata	0.0191
Trichosporon sp.	0.870
Kluyveromyces marxianus	0.0191
Antrodia xantha	0.0325
Eurotiales sp.	0.0226
Class Agaricomycetes	0.0226
Candida xylopsoci	0.0191
Ceriporia purpurea	0.0191
